# Automated preoperative volumetric analysis as a marker of the seizure irritative zone

**DOI:** 10.1007/s00701-026-06983-4

**Published:** 2026-07-18

**Authors:** Dorian M. Kusyk, Stephen Jaffee, Christopher Sanfilippo, Trent Kite, Nicholas Blaney, Jenna Li, Timothy Quezada, Tyson Tragon, Alexander C. Whiting

**Affiliations:** 1 Department of Neurosurgery, Neurosciences Institute, Allegheny Health Network, Allegheny General Hospital Tower, 320 East North Avenue, Suite 208, Pittsburgh, PA 15212 USA; 2 Department of Neurology, Neurosciences Institute, Allegheny Health Network, Pittsburgh, PA USA; 3 College of Medicine, Drexel University, Philadelphia, PA USA; 4 Department of Neuroradiology, Neurosciences Institute, Allegheny Health Network, Pittsburgh, PA USA

**Keywords:** MRI Volumetrics, SEEG, Seizure network, Irritative Zone

## Abstract

**Background:**

Stereo-electroencephalography (SEEG) is increasingly utilized in surgical epilepsy evaluations, however, more robust preoperative data can aid in localizing and focusing SEEG electrode placement. Automated MRI volumetric analysis in patients with drug-resistant epilepsy may help predict aspects of the seizure network, as confirmed with invasive SEEG analysis.

**Methods:**

Pre-operative MRIs of patients undergoing SEEG investigation were analyzed using an automated volumetric platform and compared to age- and sex-matched controls. The patient’s seizure network was parsed into subcomponents based on the SEEG investigation and compared to volumetric data.

**Results:**

Nineteen patients were analyzed. On logistic regression, each one standard deviation increase in regional volume increased the odds that a region was part of the irritative zone (OR 1.36, 95% CI = [1.08, 1.71], *p* = 0.009), though this estimate should be considered exploratory given the non-independence of regions within patients. In patients with temporal lobe epilepsy (15/19), increased volume was associated with the irritative zone (OR 1.49, CI = [1.16,1.93], *p* = 0.002) and the seizure network overall (OR 1.31, CI = [1.04, 1.65], *p* = 0.023), while decreased volume was suggestive of non-involvement with the network (OR 0.76, CI = [0.60, 0.96], *p* = 0.023).

**Conclusions:**

Regions with an increased cortical volume as determined by rapid, automated volumetric analysis were more likely to be part of the seizure network irritative zone. This may represent a novel avenue for a low-cost, non-invasive analysis of patients’ seizure networks. Further work is needed to better elucidate the relationship between cortical volumes and seizure networks and determine the potential of using automated volumetric analysis as a non-invasive tool in the preoperative work-up.

## Introduction

Epilepsy is a condition that affects about 1% of Americans per year and one third of patients with epilepsy have drug resistant epilepsy (DRE), a disease that may benefit from surgical intervention including resections, ablation and neuromodulation [13]. Surgical candidates often undergo phase I and phase II monitoring including invasive and non-invasive electrocorticography (EEG) to ensure accurate and precise description of the seizure onset zone (SOZ) [5, 6, 11, 20].

SEEG is an invasive surgical procedure involving placement of electrodes directly into the brain in order to help characterize a patient’s SOZ. Utilizing an SEEG-identified SOZ in order to guide surgical treatment of DRE can result in improved surgical outcomes [8, 14, 18, 19]. Multidisciplinary epilepsy teams utilize a combination of semiology, advanced imaging, and non-invasive EEG data to help determine successful and economic electrode implantation strategies, as each additional electrode placed carries surgical risk. Supplementary non-invasive tools are critical to refine the preoperative hypothesis, reduce electrode implantation, focus implantation in the most high-yield regions of interest and ultimately increase the likelihood of accurately identifying the SOZ.

Among non-invasive tools, magnetic resonance imaging (MRI) has been helpful for localization of focal abnormalities in patients with DRE. Specific sequences have been designed for easier identification of focal dysplasia or hippocampal pathology, and, more recently, automated volumetric analysis has been studied as a potential tool to predict components of epilepsy networks [16, 22, 23]. Automated volumetric analysis using atlas-based segmentation techniques can help identify cortical anomalies and even structural changes in anatomical regions, and the quantification of irregularity or asymmetry in hippocampi may correlate with temporal lobe epilepsy phenotypes [4, 9]. Furthermore, volumetric MRI sequencing has been used for identification of focal cortical dysplasias, demonstrating the benefit of this modality for the detection of lesions in epilepsy patients [3, 21].

Given the ability to detect subtle, abnormal tissue patterns potentially undetectable to the human eye, we postulate that automated volumetric analysis of preoperative MRIs may help better define or delineate a patient’s seizure network prior to surgical intervention. Specifically, we hypothesize that this imaging modality may help to determine if regional cortical volume changes correspond with the SOZ, propagation zone, or the irritative zone of the patient’s seizure network.

## Methods

We conducted a retrospective review of all patients who underwent SEEG at our institution from September 2021 to March 2023. Exclusion criteria included age < 18 years old and > 95 years of age. Patients with prior resections were not excluded from the sample, since the resection spaces were not sampled by SEEG, and hence the volumes which may be skewed by the resection were already not included in the analysis. The electronic medical record was queried for demographic data, medical and surgical history, and prior epilepsy workup (Table 1).

**Table 1:** Demographics of patients

Characteristic	Total
Mean Age	37.6 (20–57)
Female	5 (26%)
Prior Cranial Surgery	3 (16%)
Number of Anti-Seizure Medications	3 (1–4)
Lesion noted on MRI imaging	3 (16%)
Seizure Focus on Phase I Study	
*1. Unilateral Temporal*	12 (63%)
*2. Unilateral Frontal*	1 (5%)
*3. Multifocal*	5 (26%)
*4. Non-Lateralizing*	1 (5%)
Seizure Focus on Phase II Study	
*5. Unilateral Temporal*	10 (53%)
*6. Bilateral Temporal*	5 (26%)
*7. Frontal*	3 (16%)
*8. Multifocal*	1 (5%)
Number of Implanted SEEG electrodes	13 (9–16)
Bilateral Seizures on SEEG	10 (53%)

MRI scans were analyzed with the Icometrix ® Icobrain TBI protocol (Icometrix, Leuven, Belgium). Images were automatically segmented into cortical regions of interest in accordance with Desikan et al. [7] Volumes were then compared to a normative population (consisting of over 1500 males and over 1200 females aged between 18 and 100 from 18 different geographic datasets) via a sliding window scale of 10 years to account for any non-pathological age-related volume changes. Regional volumes were normalized for head size within the Icobrain pipeline and expressed as age- and sex-specific normative percentiles; absolute volumes were not separately corrected for total intracranial volume in this analysis.

The post-implantation high resolution computed tomography (CT) scans were fused with the preoperative MRIs using the Visage 7.1 (Visage Imaging, Inc. San Diego, CA, USA). The contacts were anatomically labelled utilizing the segmentation described by Desikan et al., to align with the terminology used by the volumetric analysis software. Based on the synthesis of the contact anatomic labeling and the SEEG record, each anatomic region was labeled as part of the seizure network: (1) SOZ, (2) early propagation zone, (3) late propagation zone, (4) irritative zone, (5) not involved, or (6) not sampled.

**Fig. 1:**
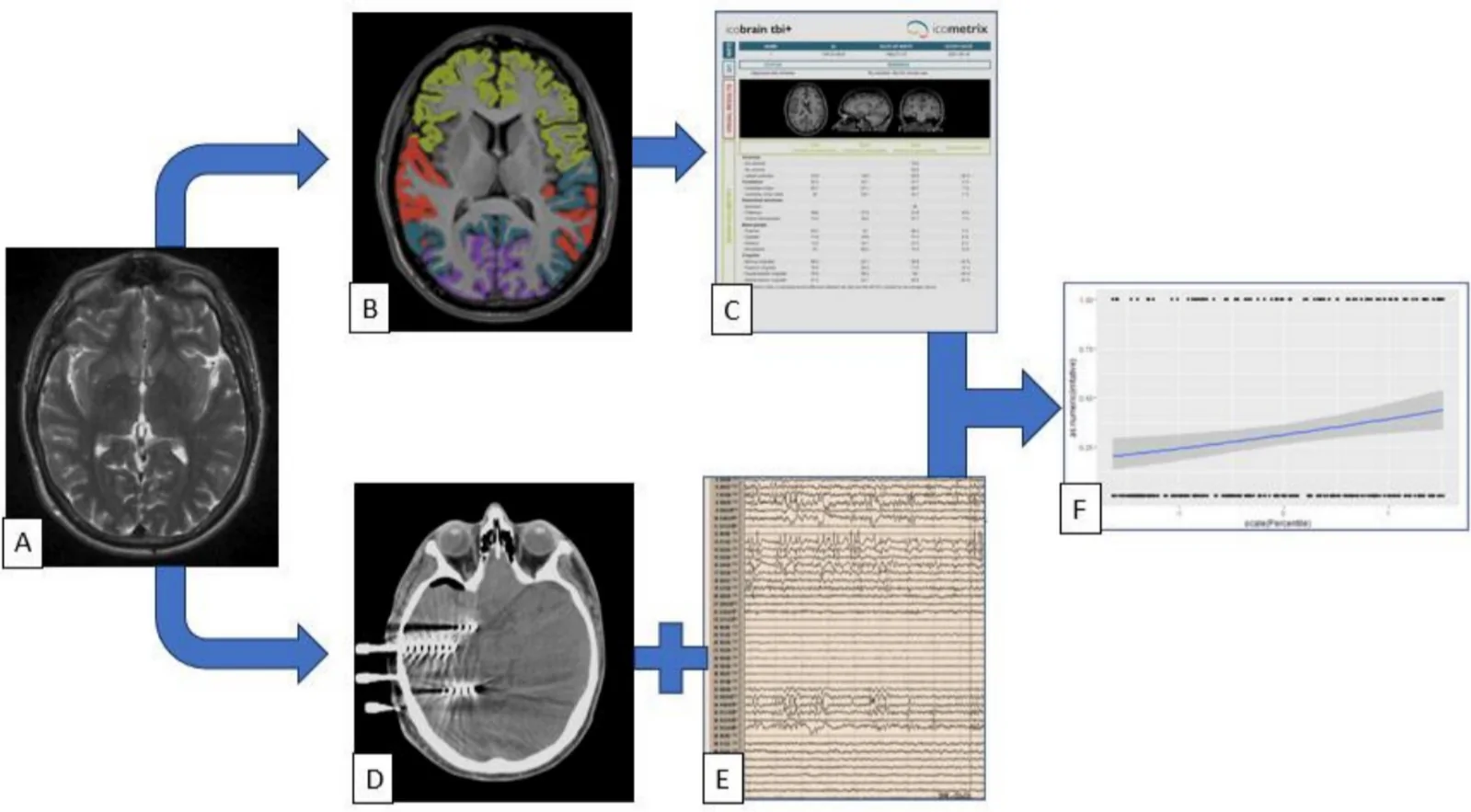
Graphical description of study design. Clockwise, Preoperative MRI (**A**) underwent automated morphometry with Icometrix. © software (**B**) which resulted in normative volumes for each of the segmented regions (**C**). The preoperative MRI was also merged with the post-SEEG implantation high resolution CT utilizing Visage 7.1 software (**D**). The contacts were manually labeled anatomically according to the definitions set forth by Desikan et al., to ensure consistent anatomic labeling between the manual process and the automated process by Icometrix. Additionally, these contacts were labeled according to their role in the seizure network after review of the SEEG record (**E**). The normative volumes generated by the Icometrix software (**C**) were then analyzed via logistic regression to see if they were predictive of seizure network involvement (**F**)

SOZ was defined as the electrographic origin of a seizure. Early propagation was defined as within 10 s of seizure onset, and late propagation was any activity after 10 s but prior to generalization. The 10-s threshold was selected a priori to separate early, more localizing spread from later, more diffuse propagation, consistent with prior network-based analyses in which the timing of seizure spread carries localizing value [1, 3]. An irritative zone was defined as the presence of interictal discharges. Regions not identified in the previous four roles were labeled as not involved in the seizure network. A summary of the analysis pipeline is described in Fig. 1.

Statistical analysis was performed with the R package (www.r-project.org) version 4.2.0. For analysis, the normative volume percentile was converted from a 0–100 scale to a centered standard deviation range of −2 to 2. A logistic regression model was used to evaluate whether centered volume percentile was associated with any of the elements of the seizure network. Subgroup analysis based on seizure subtype (temporal lobe epilepsy vs extra-temporal epilepsy) was also performed. This study was approved by the Institutional Review Board (AHN-IRB 2021–314).

## Results

Our cohort consisted of nineteen total patients (*n* = 19), 26% were female (*n* = 5), mean age was 38 years old (range, 20–57), and patients were maintained on an average of three anti-seizure medications (range, 1–4). Regarding prior surgical history, 11% of patients (*n* = 2) had Vagus nerve stimulators, 11% (*n* = 2) had prior right anterior temporal lobectomies, and 5% (*n* = 1) had a hippocampal laser interstitial thermal ablation. 16% of patients (*n* = 3) had mesial temporal sclerosis on MRI, the remaining 84% (*n* = 16) of patients had non-lesional epilepsy on MRI. Detailed histopathologic subtyping (e.g., focal cortical dysplasia) was not available for the non-lesional cohort, and the small number of lesional cases (three with mesial temporal sclerosis) precluded a statistically meaningful comparison of volume effects across individual etiologies.

95% (*n* = 18) of patients underwent bilateral SEEG implantation during phase II study. The average number of electrodes implanted per patient was 12.6 (range of 9–16). In total, 239 electrodes were implanted, 54% (*n* = 128) on the right and 46% (*n* = 111) on the left. Electrodes were found to sample 408 total anatomic regions as defined by the anatomic segmentation presented in Desikan et al. [7]The left hippocampus was targeted in 95% of our patients (*n* = 18). 58 unique regions were sampled within our cohort. 38% (*n* = 154) regions were noted to be involved in a seizure network, 14% (*n* = 56) of regions were found to have multiple roles in the seizure network. 11% (*n* = 43) of regions were noted to be part of the SOZ, most commonly the left hippocampus (*n* = 8), followed by the right hippocampus, right amygdala, and left insula (*n* = 4). The breakdown of the SEEG data can be found in Table 2.

**Table 2:** Summary data from SEEG

Total Regions Sampled	Total
*1. Seizure Onset Zone*	43 (11%)
*2. Early Propagation Zone*	29 (7%)
*3. Late Propagation Zone*	30 (7%)
*4. Irritative Zone*	111 (27%)
*5. Regions with Multiple Roles in Seizure Network*	56 (14%)
*6. Not Involved in Seizure Network*	254 (62%)

**Fig. 2:**
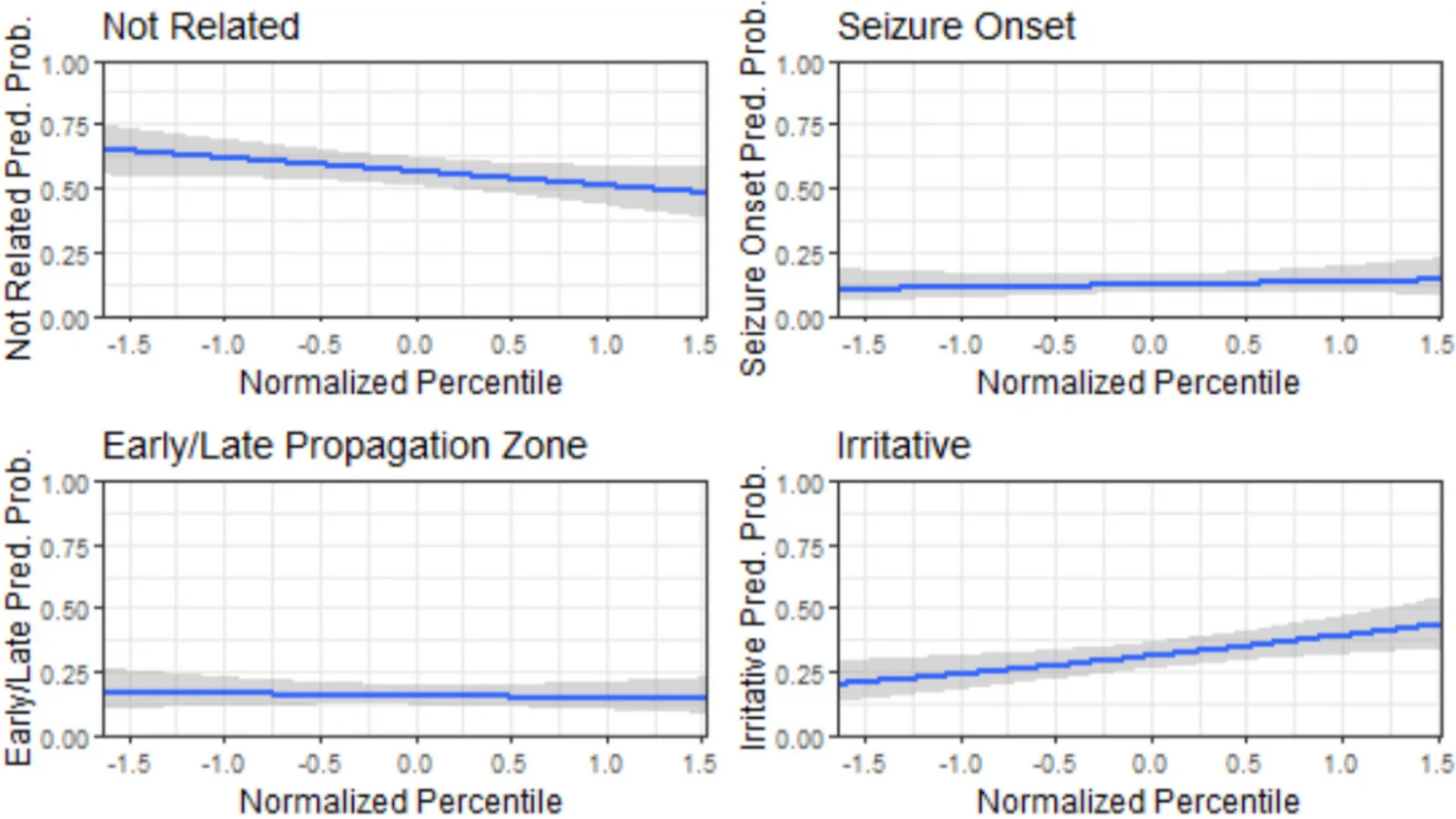
Outcome predicted probability by normalized percentile. The irritative zone was the only test that provided consistent statistical significance, while the regions described as “not related” to the seizure network trended towards a significant association with smaller cortical volumes. Neither Seizure Onset zones nor Early/Late Propagation zones were found to be significantly associated with regional volume in our cohort

Volumetric analysis did not predict SOZ, early or late propagation zones (Table 3). Logistic regression modeling of all patients demonstrated a statistically significant relationship between normative volumes and irritative zones (i.e. regions not also labeled as a SOZ or propagation zone during another seizure) with an odds ratio of 1.36 (95% CI = [1.08, 1.71], Fig. 2); because regions were clustered within patients, this odds ratio and its confidence interval should be interpreted as exploratory. In the temporal lobe epilepsy subgroup (*n* = 15), increased regional volume was positively associated with the irritative zone (OR 1.49, 95% CI = [1.16,1.93], *p* = 0.002). Additionally, regional volume was negatively associated with areas outside of the seizure network for patients with only temporal lobe epilepsy (OR 0.76, 95% CI = [0.60, 0.96], *p* = 0.023) (Table 4).

**Table 3:** Logistic regression model of all patients

	Odds Ratio (95% CI)	*p*-value
Seizure Onset Zone	1.08 (0.79, 1.50)	0.621
Early Propagation Zone	0.97 (0.66, 1.43)	0.884
Late Propagation Zone	0.90 (0.62, 1.31)	0.572
**Irritative Zone**	**1.36 (1.08, 1.71)**	**0.009**
Any involvement in seizure network*	1.23 (1.00, 1.52)	**0.048**
Not implicated in seizure network	0.81 (0.66, 1.00)	**0.048**

Bolded cells highlight statistical significance. * “Any involvement in the seizure network” defined as seizure onset zone, a propagation zone or irritative zone

**Table 4:** Logistic regression model of only patients with temporal lobe epilepsy

	Odds Ratio (95% CI)	*p*-value
Seizure Onset Zone	1.31 (0.92, 1.91)	0.142
Early Propagation Zone	0.91 (0.61, 1.37)	0.66
Late Propagation Zone	0.67 (0.42, 1.05)	0.089
Irritative Zone	**1.49 (1.16, 1.93)**	**0.002**
Any involvement in seizure network*	**1.31 (1.04, 1.65)**	**0.023**
Not implicated in seizure network	**0.76 (0.60, 0.96)**	**0.023**

Bolded cells highlight statistical significance. * “Any involvement in the seizure network” defined as seizure onset zone, a propagation zone or irritative zone

## Discussion

In this manuscript, we found that the increased volume of a cortical region correlated with increased odds that the region was part of the seizure network’s irritative zone. Furthermore, this association was magnified when looking at regions which were purely irritative zones and were not also noted to be either seizure onset zones or propagation zones. Regions which were sampled but did not have a role in the seizure network were more likely to have smaller volumes on analysis and were more likely to have smaller volumes on analysis in the patients diagnosed with temporal lobe epilepsy (*n* = 15).

In this study, we attempted to use novel automated volumetric analysis of patient MRI scans to predict local regional involvement in seizure networks. There has been significant investigation into the use of MRI volumetric analysis for epilepsy detection. Hakyemez et al. [10] conducted volumetric analysis of patients with temporal lobe epilepsy (TLE) compared to a matched control group to assess limbic structures including the amygdala, hippocampus, mamillary bodies and fornixes. Control group patients had statistically significant difference in mammillary bodies, fornixes and hippocampi compared to the TLE group, there was no statistically significant difference in the size of amygdala. This investigation conducted in 2006 highlighted the potential for use of MRI for differentiating non-lesional epilepsy patients from the general population [10].

Moreover, Singh et al. investigated patients with non-lesional temporal lobe epilepsy and found statistically significant amygdala enlargement in their volumetric analysis in 16% of their patients, of those with amygdala enlargement, 88% of which had amygdala enlargement on the same side as their epileptogenic zone localization [21]. Iqbal et al. conducted further study in this field and demonstrated no statistically significant difference in the size of the piriform cortex amongst TLE and controls. Notably, patients with hippocampal sclerosis also had reduced piriform cortex volumes [12].

Efforts have also been made to detect temporal lobe epilepsy with the combination of volumetric analysis and PET scan. Aslam et al. analyzed volumetric MRI and 18F fluorodeoxyglucose PET asymmetry. They found a high sensitivity and specificity related to an approximate 4% reduction in hippocampal volume with less specificity and sensitivity for the use of PET scan. This study demonstrates the versatility of imaging modalities for epilepsy detection but highlights the potential reliability of volumetric analysis within this space [2].

To our knowledge, this is one of the first volumetric analyses of seizure networks compared directly to patients’ SEEG data. Whiting et al., looked at similar automated volumetric morphometry in patients who underwent frontal lobectomies and found that volume loss, even outside of the frontal lobe resection, was predictive of poor seizure outcomes [22]. However, though their volumetric analysis used similarly granular anatomic regions, the authors were unable to comment on the seizure network itself. They did hypothesize that volume loss distant to the frontal lobe was suggestive of a more diffuse seizure network. Likewise, Manmatharayan et al., utilized automated volumetric analysis of the temporal lobe in patients with mesial temporal sclerosis, and found that volume loss across various mesial temporal structures was predictive of continued seizures after intervention [15].

Cortical region hypertrophy, seen within irritative zone, has not been reported as frequently as regional atrophy. Neuhaus et al., did note that patients with epilepsy displayed higher gray matter volume in both the amygdala and the parahippocampal gyri [16]. It is possible that the hypertrophy seen in the irritative zones in our cohort is a sign of local irritation from interictal activity. However, it is unclear whether irritative zones in untreated epilepsy patients ultimately progress to atrophy as they become more involved in the seizure network without further investigation into the natural progression of these volume changes. Several explanations merit consideration. Biologically, increased regional volume could reflect interictal hyperexcitability with associated gliosis, inflammation, or activity-dependent structural remodeling, or it could represent a primary developmental abnormality such as subtle cortical dysplasia that is itself epileptogenic. Methodologically, atlas-based segmentation can over-estimate the volume of regions with blurred gray–white junctions or subtle dysplastic change, so apparent hypertrophy may in part reflect segmentation behavior rather than true tissue excess. Our cross-sectional design cannot distinguish these possibilities, and the directionality we observed should be interpreted with caution given the atrophy reported in many epileptogenic regions.

SEEG implantations are now frequently being utilized as the gold-standard for defining a patient’s epilepsy network prior to surgical intervention meant to achieve seizure control or freedom, particularly in patients with non-lesional epilepsy or discordant non-invasive data. The goal of a phase I evaluation prior to SEEG is to collect as much information as possible in a non-invasive and comprehensive manner that may help better delineate or define the patient’s epilepsy network. A less-precise preoperative hypothesis may require a more extensive implantation strategy, with additional electrodes placed. While the overall risk of SEEG electrode implantation is low, each additional electrode does carry the small, but potentially devastating risk of intracranial hemorrhage. The goal of this study was to investigate a novel tool to potentially help better define or focus the preoperative hypothesis. High quality MRIs are part of the routine work-up of pre-surgical DRE patients, so automated, volumetric analysis would be a relatively simple and cost-efficient addition to phase I evaluations if proven efficacious in further helping to define potential epilepsy networks.

Though it is one of the largest series of SEEG patients with concurrent volumetric morphometry, it remains a relatively small cohort of patients. Due to the small cohort size, we were unable to control for all confounding patient characteristics. For example, some anti-seizure medications have been noted to increase cortical thickness and our cohort consisted of mainly temporal epilepsy patients who had discordant findings on non-invasive workup to necessitate a SEEG investigation [17]. Two further limitations warrant emphasis. First, because multiple anatomic regions were analyzed per patient, these regions are not strictly independent observations; clustering of regions within patients may bias the variance estimates, and the present odds ratios should therefore be regarded as exploratory rather than definitive. Second, the significance of an increased cortical volume likely differs by underlying etiology — mesial temporal sclerosis, amygdalar enlargement, and focal cortical dysplasia each carry distinct volumetric signatures — and our cohort was too small and etiologically heterogeneous to model these effects separately.

We compared normative volumes to seizure network involvement as defined by SEEG. As such, we cannot definitively prove that a patient’s seizure onset zone was both necessary and sufficient to provoke seizures, since several patients did not undergo surgical resection for their epilepsy and underwent neuromodulation surgery. Further work is needed to identify if either resection or neuromodulation of the irritative zones improves patient outcomes. Additionally, the SEEG implantation itself introduces an element of sampling bias – since most of the cohort had findings of temporal lobe epilepsy, the implantations were heavily skewed towards temporal regions.

Finally, it is important to contextualize these findings within the framework of the epileptogenic zone. The irritative zone, defined by interictal discharges, is not synonymous with the seizure onset zone or the epileptogenic zone and is frequently more extensive than the region whose removal is required for seizure freedom. We therefore do not propose volumetric analysis as a stand-alone means of identifying resection targets. Rather, regions of increased cortical volume may serve as supplementary, non-invasive flags that help refine the preoperative hypothesis and prioritize electrode coverage during SEEG planning, particularly in non-lesional cases where implantation relies on converging lines of indirect evidence. The clinical value of this approach will ultimately depend on prospective validation against post-resection seizure outcomes.

## Conclusions

Automated volumetric morphometry in temporal lobe epilepsy may have a role in the pre-operative planning of surgical epilepsy patients and these methods may be used to inform a more targeted SEEG implantation. Prospective studies incorporating volumetric analysis with comparison of epileptogenic and non-epileptogenic regions will be needed to further validate this imaging modality for targeting irritative zones and surrounding regions. As a marker of the irritative zone rather than the seizure onset zone, automated volumetric analysis is best positioned as a supplementary input to the preoperative hypothesis rather than a stand-alone determinant of resection targets.

## Data Availability

The datasets generated and analyzed during the current study are not publicly available but are available from the corresponding author upon reasonable request.

## Abbreviations

SEEG: Stereoelectroencephalography
EMU: Epilepsy Monitoring Unit
